# Smartphones for musculoskeletal research – hype or hope? Lessons from a decennium of mHealth studies

**DOI:** 10.1186/s12891-022-05420-8

**Published:** 2022-05-23

**Authors:** Anna L. Beukenhorst, Katie L. Druce, Diederik De Cock

**Affiliations:** 1 Department of Biostatistics, Harvard T.H. Chan School of Public Health, 677 Huntington Avenue, Boston, MA 02115 USA; 2 Centre for Epidemiology Versus Arthritis, University of Manchester, Manchester Academic Health Science Centre, Manchester, UK; 3 Skeletal Biology and Engineering Research Center, Department of Development and Regeneration, KU Leuven, Leuven, Belgium

**Keywords:** mHealth, Musculoskeletal diseases, Smartphones, Digital health, Mobile health, Chronic pain, Sensor data, Patient-reported outcomes, Engagement, Passive data, eHealth

## Abstract

**Background:**

Smartphones provide opportunities for musculoskeletal research: they are integrated in participants’ daily lives and can be used to collect patient-reported outcomes as well as sensor data from large groups of people. As the field of research with smartphones and smartwatches matures, it has transpired that some of the advantages of this modern technology are in fact double-edged swords.

**Body:**

In this narrative review, we illustrate the advantages of using smartphones for data collection with 18 studies from various musculoskeletal domains. We critically appraised existing literature, debunking some myths around the advantages of smartphones: the myth that smartphone studies automatically enable high engagement, that they reach more representative samples, that they cost little, and that sensor data is objective. We provide a nuanced view of evidence in these areas and discuss strategies to increase engagement, to reach representative samples, to reduce costs and to avoid potential sources of subjectivity in analysing sensor data.

**Conclusion:**

If smartphone studies are designed without awareness of the challenges inherent to smartphone use, they may fail or may provide biased results. Keeping participants of smartphone studies engaged longitudinally is a major challenge. Based on prior research, we provide 6 actions by researchers to increase engagement. Smartphone studies often have participants that are younger, have higher incomes and high digital literacy. We provide advice for reaching more representative participant groups, and for ensuring that study conclusions are not plagued by bias resulting from unrepresentative sampling. Costs associated with app development and testing, data storage and analysis, and tech support are substantial, even if studies use a ‘bring your own device’-policy. Exchange of information on costs, collective app development and usage of open-source tools would help the musculoskeletal community reduce costs of smartphone studies. In general, transparency and wider adoption of best practices would help bringing smartphone studies to the next level. Then, the community can focus on specific challenges of smartphones in musculoskeletal contexts, such as symptom-related barriers to using smartphones for research, validating algorithms in patient populations with reduced functional ability, digitising validated questionnaires, and methods to reliably quantify pain, quality of life and fatigue.

## Background

The worldwide impact of around 200 musculoskeletal conditions that affect joints, bones, muscles and soft-tissues, is large [1–4]. Many musculoskeletal conditions are characterized by pain, stiffness, fatigue, sleep disturbances [5]. These symptoms affect patients’ physical function andimpede activities of daily living [4, 5]. Although the past century has seen advances in diagnosis accuracy and treatment options, people with musculoskeletal conditions still face high impact of disease, even if they achieve clinical remission [1–3].

To improve patients’ quality of life, a better understanding of fluctuations in disease state, symptoms and wellbeing over time is crucial, also in people with clinical remission. Smartphones could provide opportunities to better understand these fluctuations in patients’ everyday lives [6]. In addition, smartphones can enable monitoring, reporting of patient-reported outcomes, better patient-provider communication and personalized treatments [7].

The promise of smartphones in musculoskeletal research and clinical care has been widely recognized. Various studies and publications [6–24] have documented key benefits of smartphone data collection for both research and clinical care, namely that it:

1. Offers opportunities to answer research questions that were difficult to investigate before.
2. Allows recruitment and engagement of large groups of participants with a range of musculoskeletal conditions, increasing power to detect associations
3. Supports the relatively unobtrusive collection of real-time, high frequency data for measuring exposures, outcomes and behaviour, ranging from daily to multiple times per millisecond.
4. Can reduce burden of providing patient-reported outcomes and sources of reporting bias.
5. Can identify recurring patterns in the symptoms of individual participants, detect real-time deviations from this baseline and aggregate these to summarize populations
6. Augment our understanding of patients’ lived experiences by taking more frequent measurements and/or combining key symptom domains, disease characteristics and behavioural patterns
7. Can support the development, and eventually delivery, of patient-centred and personalized care.

Smartphones and mobile health have been hailed as ‘the biggest technology breakthrough of our time’ with ‘the potential to change every aspect of the health care environment’ [25]. Despite success stories, smartphone studies have not delivered on all promises, and are certainly are no panacea to all limitations of traditional data collection methods [18]. Researchers should recognize various novel challenges for data collection and analysis, as well as various sources of bias [25].

.

After a decade of smartphone research, the research community has gained a better understanding of opportunities and challenges in musculoskeletal research [7, 26–29]. At the same time, research groups and commercial partners continue developing bespoke apps, potentially ‘reinventing the wheel’. In addition, various pitfalls of smartphone studies have been discovered in other medical application domains, but are not necessarily known to the musculoskeletal research community. In this narrative review, we discuss smartphone studies in musculoskeletal research and outline the advantages of smartphones for data collection. We highlight common misconceptions about smartphones, link these to pitfalls of smartphone research, and discuss our hope for overcoming these pitfalls.

Of note, this paper’s scope is limited to the use of *smartphones* for musculoskeletal research. Some of the challenges and opportunities we discuss will be applicable on technology more widely (e.g. on wearables, web portals or mobile health in general). However, we did not actively include literature on these types of technology in this article. Similarly, this article does not focus on the use of smartphones in clinic to support shared decision-making, although some of the aspects we discuss generalize to that setting [6, 30].

## Main text

### Smartphones for musculoskeletal research

As smartphones are ubiquitous and typically carried by their users, they can be used for unobtrusive measurement of frequent active and passive data [31, 32]. Active data include all data types that require input from the participant, such as smartphone surveys, audio recordings, photos or ‘active tasks’ (e.g. tapping the screen as fast as possible).

Passive data include all data types that do not require participant action beyond installing a data collection app, such as sensor data and metadata [27]. Smartphones typically have the following in-built sensors: GPS receivers, accelerometers, gyroscopes, magnetometers, microphones, barometers, digital compass, and proximity and light sensors [33–37]. These sensors can be used to measure user behaviour and environment, including factors such as:

- exposure to pollution or weather (e.g. after linkage of location data to weather databases individual-level [17, 38]),
- mobility (e.g. distance travelled, travel patterns, maximum distance from home, visits to health clinics [39]),
- routines (diurnal movements, travel routines, out-of-home activities, interactions with other people),
- physical activity (walking, standing, running, sitting, falling, mode of transportation, gait speed, intensity [36, 40])
- ambient environment (light, presence of people), and
- sociability (locations visited, vicinity to other smartphone users)

Passive data collection may also refer to the accrual of metadata. Metadata refers to digital information on how the device or app is used. These include timestamps for tasks including survey completion, passive data, call and text logs (timing, duration/length of incoming and outgoing calls and texts), battery status and phone charging events [41]. Metadata can be useful to validate data by determining the timing of self-report entries, compared to the completion time requested. However, metadata also offers unique opportunities to collect information about participants’ behaviour such as screen use, sociability or help seeking (as measured by call and text logs), circadian rhythm (determined based on the timing of activities throughout the day) and engagement with a digital intervention (as measured by app use) [27, 39].

Table 1 shows 18 examples of studies in people with musculoskeletal conditions ranging from rheumatoid arthritis and osteoarthritis to juvenile idiopathic arthritis and systemic lupus erythematosus. We included 10 examples of observational studies and 8 examples of interventional studies, to showcase studies across a range of musculoskeletal conditions, collecting different types of outcome metrics. Of note, for this narrative review we did not perform a systematic search of app stores or the academic literature, but we merely collected examples that showcased the advantages and pitfalls of smartphone research well. A systematic review is a great method to identify smartphone apps or smartphone studies and appraise their quality. We refer readers who are interested in such systematic reviews to reviews published here and elsewhere, including a review (1) rating the quality of 19 apps to monitor rheumatoid arthritis [50], (2) describing one study using a smartphone app [51], (3) identifying 4 apps to facilitate physical activity in people with rheumatoid arthritis [52], (4) identifying 20 apps for people with systemic lupus erythematosus [28], (5) describing 61 apps on self-managing low back pain [53], and (6) providing an overview of development of 32 apps for self-management of musculoskeletal diseases [54]﻿.

**Table 1:** Scoping review of smartphone studies, by year of publication and then by alphabetical order of first author name

Author, year (ref)	Study name/acronym	Study type (obvs/int)	Aim	Population (*n* =)	Follow-up	PROs	Active Tasks	Wearable
Lalloo et al., 2021 [9]	iCanCope	Intervention	Improve coping/self management	Adolescents with JIA (*n* = 60)	8 weeks	X	X	
Nap-van der Vlist et al., 2021 [11]	ProFeel	Intervention	Reduce fatigue	Fatigued adolescents with chronic conditions (*n* = 57)	6 weeks	X	X	
Nowell et al., 2021 [12, 13]	ArthritisPower app	Observational	Feasibility to inform use of future RA study design	Rheumatoid arthritis (*n* = 253)	3 months	X	X	
Khan et al., 2020 [10]	NA	Intervention	Improve self-management	Systemic lupus erythematosus (*n* = 50)	16 weeks	X	X	
Lee et al., 2020	MyPainTracker	Observational	Measure pain location	Juvenile idiopathic arthritis (*n* = 14)	8 weeks	X		
Rodríguez-Sánchez-Laulhé et al., 2020 [14]	Carehand	Intervention	Improve hand function	Hand Rheumatoid arthritis (*n *= NR)	3 months		X	
Van der Veer et al., 2020 [42]	Pain manikin	Observational	Measure pain location and intensity	Any musculoskeletal condition (*n* = 8)	NA	X		
Austin et al., 2019 [6]	REMORA	Observational	Report disease activity and impact	Rheumatoid arthritis (*n* = 20)	3 months	X		
Beukenhorst et al., 2019 [22, 43]	KOALAP	Observational	Link between exercise and pain	Knee osteoarthritis (*n* = 26)	90 days	X		X
Dixon et al., 2019 [17, 44, 45]	Cloudy with a Chance of Pain	Observational	Effect of weather on pain	Chronic pain (*n* = 10,584)	15 months	X		
Gossec et al., 2019 [24]	ActConnect	Observational	Predict disease flares	Rheumatoid arthritis / axial spondyloarthritis (*n* = 155)	3 months	X		X
Tam et al., 2019 [16]	OPERAS	Intervention	Improve self-management	Rheumatoid arthritis (*n* = 134)	6 months	X	X	X
Crouthamel et al., 2018 [46, 47]	PARADE	Observational	Feasibility of measuring functional ability	Rheumatoid arthritis (*n* = 399)	12 weeks	X	X	
De la Vega et al., 2018 [48]	Fibroline	Observational	Feasibility of digital CBT	Fibromyalgia (*n* = 29)	9 weeks	X	X	
Druce et al., 2018 [21]	QUASAR	Observational	Investigate sleep quality and QoL	Rheumatoid arthritis (*n* = 350)	30 days	X		X
Mollard et al., 2018 [49]	LiveWith Arthritis	Intervention	Improve self-management and health outcomes	Rheumatoid arthritis (*n* = 21 app users; *n* = 15 controls)	6 months	X		
Day et al., 2017 [8]	Healthy.me	Intervention	Improve control over serum urate levels	Gout (*n *= NA)	6 months	X	X	
Skrepnik et al., 2017 [15]	OA GO	Intervention	Improve mobility	Osteoarthritis (*n* = 211)	90 days	X	X	X

*AxSpa* Axial Spondyloarthritis, *FM* Fibromyalgia, *JIA* Juvenile Idiopathic Arthritis, *NA* Not Applicable*, NR* Not Reported,* OA* Osteoarthritis, *PROs* Patient-Reported Outcomes, *RA* Rheumatoid arthritis, *RMDs* Rheumatic Musculoskeletal Diseases, *SLE* Systemic Lupus Erythematosus, *QoL* Quality of Life

The intervention studies in Table 1 focused on improving self-management [9, 10, 15, 48, 49], or targeted more specific outcomes including mobility [16], hand function [14], fatigue [11], pain [49], physical function [49], or serum urate levels in a gout population [8].

The observational studies often examined environmental exposures or behaviour, including weather [17] or exercise [22, 43], investigated pain location and intensity [22, 42, 43, 55], functional ability [46, 47], disease flares [24] or disease activity and its impact [6]. Various studies were pilot studies or feasibility studies [22, 43, 46–48].

Ten studies asked participants to perform “active tasks”, a predefined activity that provides insight in physical functioning, cognition or motor skills. The active tasks used in these papers included: a wrist motion test [46], yoga exercises and cognitive behavioural therapy exercises [48], obtaining and entering serum urate levels [8], a walking exercise [15, 46], performing behavioural interventions chosen by a health coach [10, 14, 16], sharing experiences by answering questions from other participants [9], or active tasks chosen by the participants [11–13]. One study asked patients to upload a photo of their hand once a month, which was used to monitor inflammation and deformity [49]. Five studies showcase how data from smartphone apps can be combined with data from a wearable. Four projects used it for sensor data collection only [15, 16, 21, 24] and one for both sensor data and patient-reported outcomes [22, 43].

These studies demonstrate some of the advantages of using smartphones for data collection. First, frequent measurements increase the power of studies to detect associations. Four studies recruited hundreds of participants, showcasing the advantage of remote recruitment and lower marginal effort per newly recruited participant [12, 13, 17, 21, 44, 46, 47]. The included studies were able to collect data frequently for sustained periods of time, ranging from 30 days to 15 months. In addition, high frequency longitudinal data provided insights into day-to-day fluctuations in symptoms, and their impact on activities of daily living and quality of life [6]. This resulted, for example, determination of thresholds for flares in axial spondyloarthritis [24].

Moreover, smartphones enable more detailed patient-reported outcomes. One study collected the patient-reported outcomes to guide subsequent visits to a rheumatologist [6]. Three studies used digital pain manikins to enable participants to locate pain, and specify both the extent of pain and the intensity [42, 46, 47]. Previously, musculoskeletal researchers highlighted the simplicity of existing pain trackers for smartphones, and the need for more rigorously tested apps that record location-specific pain aspects and convert data into quantitative scores for pain extent and intensity [29, 56].

For intervention studies, the integration of smartphones in participants’ daily lives provided an opportunity to influence behaviour by sending unobtrusive, context-aware messages at the right place at the right time. This was used in various studies aiming at improving function, mobility, self-management or coping behaviour [8–11, 14–16].

Second, smartphones offered the opportunity to collect passive data on real-life behaviour, exercise regimen and environmental exposures via sensors [17, 27, 31]. Sensor data is free from the biases associated with self-reported data, such as social desirability bias and recall bias [57]. The reviewed studies used sensor data to determine location where participants were exposed to the weather [17], or to quantify their mobility and physical functioning [15, 22].

Third, participants used their own device for data collection and even enrol in studies online. In contrast to paper-based diaries or research-grade wearables, data transfer did not require returning diaries or devices to trained research staff. Instead, data transfer was done automatically, using WiFi or cellular connection. This advantage allowed for recruitment of large sample sizes [17], large sample sizes relative to the prevalence of a musculoskeletal condition [10], or collection of large quantities of data [11–13, 24, 43].

### The hype, the reality and the hope

**Fig. 1:**
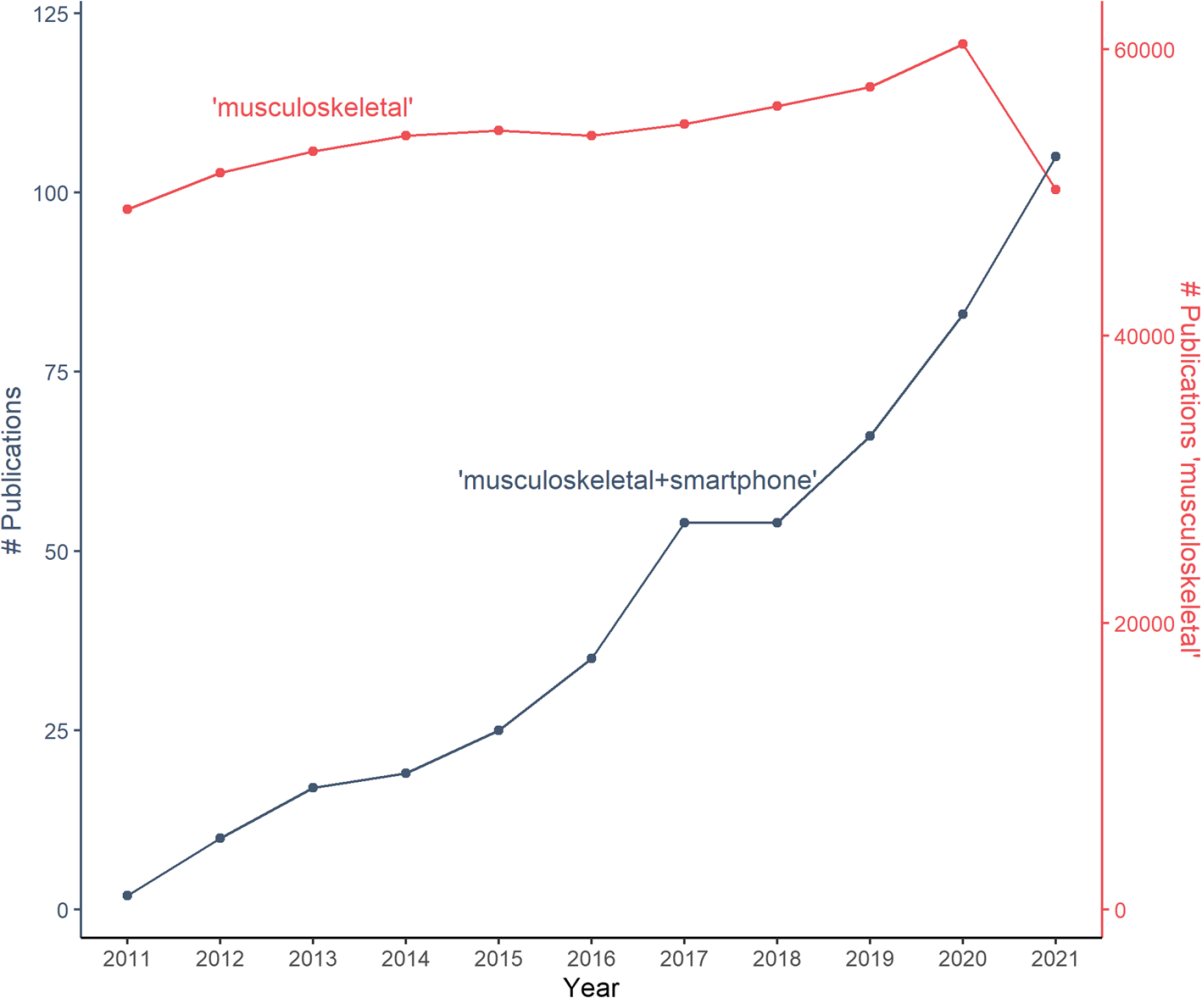
Between 2011 and 2021, the number of Pubmed-indexed publications with search term ‘musculoskeletal’ and ‘smartphone’ increased sharply from 0 in 2011 to 105 in 2021. In the same period, publications with search term ‘musculoskeletal’ only increased gradually, with drops in 2016 and 2021

Enthusiasm for using smartphones as tools for data collection has skyrocketed (Fig. 1). Given the tendency to focus on advantages of smartphone studies only, it may even border on a hype. Many of characteristics of smartphones are in fact double-edged swords: they provide advantages compared to traditional data collection methods, but also impose new challenges. For appropriate use of smartphones and maximization of their benefits, researchers need to be aware of and account for these new challenges. We will therefore present common myths, provide a more nuanced overview of both the upsides and downsides of smartphone studies and outline what further steps are required to harness the upsides while handling the downsides.

### Hype: smartphone studies enable high engagement

Smartphones are often used to collect self-reported data and patient-reported outcomes at a larger scale. Compared to, for example, paper-based diaries, smartphone studies make it easier to recruit large sample sizes and collect more data, more frequently. Traditionally, self-reported data was collected multiple times an hour over a period of days, or, at the other end of the scale: daily self-report for months to a year [58]. Paper-based diaries typically require more effort to completion, which increases attrition. Studies using paper-based diaries often face low compliance: participants lose diaries, forget to send them back, forget to complete them, hoard and backfill paper-based surveys, or misdate survey entries [57, 59]. Hoarding, backfilling and misdating can influence study validity enormously: one study showed apparent compliance rates of 90% (based on participant-reported completion), while a hidden sensor showed it was actually 11% [60].

Compared to paper-based diaries, smartphones have a lower burden of data entry. In addition, they provide possibilities to increase compliance and validate it: they automatically timestamp and upload (or “send back”) data entries. However, high engagement, high compliance and low attrition are by no means a characteristic of smartphone studies [20, 61]. Smartphone researchers need to be aware of the challenges to engagement, attrition and compliance in smartphone study and account for these at study design and during data analysis.

### Reality: high engagement requires substantial effort and attrition is a significant threat to smartphone study validity

Although the burden of data entry on smartphones is regarded as low, this does not mean that engagement is automatically high in smartphone studies. One challenge for smartphone studies is that no universal definition of engagement exists. Engagement may refer to days of self-reported data entry, days of passive data collection (any passive data, or only above a threshold), duration of usage (i.e. time between first and last assessment), proportion of days with self-reported data entry, or more complex definitions including likelihood of data completion overtime, thresholds for a minimal amount of data collected or, for interventions, the ‘usage half-life’ [20, 43, 61–64]. This variety of definitions illustrate the complexity of describing smartphone data, which is typically frequent, and includes data from numerous data streams.

Regardless of the definition used, many studies have shown that missing data due to attrition and low engagement is common, even a characteristic of smartphone studies and smartphone interventions [61, 62, 65, 66]. Various reasons have been named for this so-called ‘law of attrition’ [61] in smartphone studies, including competing priorities, ease of dropping out, user experience and usability issues [43, 61, 66]. This illustrates the double-edged sword: although smartphones are unobtrusive and integrated in participants’ daily lives, research smartphone apps compete for attention with all those other apps and activities of daily life.

High attrition and low engagement threaten both the internal and external validity of studies. Study results may not generalize to participants that drop-out early if these dropouts systematically differ from those who stay engaged. Various studies have shown that demographic characteristics, such as age, sex and socio-economic status, are associated with the risk of drop-out [62, 67, 68]. As these characteristics are also associated with disease, disease severity, comorbidities and other outcomes, differential drop-out may introduce attrition bias. Especially in musculoskeletal diseases, participants can face additional barriers when using smartphones. For example, some previous studies found that people with rheumatoid arthritis had issues of dexterity or data entry issues when using a smartphone research app [17, 20, 21, 69].

### Hope: researchers design smartphone studies to promote engagement, reduce attrition and report attrition rates

Researchers should be aware of the challenges of engagement in smartphone studies and can use various strategies to improve engagement and reduce the risks of attrition bias. Previous studies have provided advice for better study designs and data analyses. We have grouped these in three areas relevant for all smartphone studies, and two areas relevant for interventional studies specifically.

First, researchers can increase the usability of technology. Pilot studies or feasibility studies in the target population can be useful to improve these aspects of the smartphone app or study design [21, 22, 32, 69, 70]. In healthy populations, high daily smartphone use has a weak relationship with lower hand grip strength and pinch-grip strength [71, 72]. In people with musculoskeletal symptoms and pain, especially in their hands, it is therefore important to assess their ability to navigate research apps and report symptoms. To increase engagement, researchers should integrate patients’ perspectives and needs into their study designs [7]. In addition, it can be useful to use the expertise of healthcare providers, family members (especially in the case of juvenile idiopathic arthritis [70]) and information technology experts [73]. Upon the launch of the study, researchers can monitor data collection to diagnose app or device problems early [74] and enable participants to be contacted to prevent attrition [20].

Second, researchers can reduce the active data collection, which requires the participant to enter data or do an active task, and increase sensor data collection. Studies show that sensor data is often collected for longer periods than active data like surveys, as it provides less burden to the participants [43, 75].

Third, researchers can design their study to promote engagement. They can introduce motivating factors (such as rewards or visualisations of inputted data), use both in-built reminders and targeted reminders (i.e. in the case of likely drop-out), create a study community (e.g hosted on social media sites), provide of personal support from a named member of study personnel, and, if resources allow actively target personal motivators [20]. In addition, they can provide overviews of participants’ self-reported symptoms to share with a clinician or other forms of feedback, which have been shown to increase participant interest [43, 68]. A currently ongoing randomized clinical trial is evaluating whether skipping clinic visits if smartphone data shows low disease activity is beneficial to patients [73].

Fourth, researchers should investigate engagement, attrition and its consequences when analysing their data. Researchers should not gloss over attrition rates, but report engagement and attrition metrics, show Kaplan-Meier curves and report attrition rates (more information in [61]).

Fifth, interventional studies can use recommendations from previous interventional studies using smartphones [32, 66]. These recommendations include, for example, recommendations on pilot studies, on avoiding one-size-fits-all approaches, and on reducing participants’ self-care burden. As it is not within our scope to provide an exhaustive overview of every recommendation ever done, we refer readers to those source publications.

Sixth, interventional studies can use the ‘run-in and withdrawal design’, where participants are only randomized if they are still active after a weed-out period [61]. Many interventional smartphone studies face an exponential dropout early in the study. If randomization is postponed until after this phase, the intervention and control groups are less likely to contain early dropouts. If the randomized participants stay engaged for longer, the study will have higher power and may provide a more realistic image of the intervention. Of note, results of studies using the run-in and withdrawal design may only apply to the ‘hardcore users’, i.e. participants that engaged, and may not be generalizable to the participants that dropped out early and were never randomized.

### Hype: smartphone studies reach a more representative sample

Smartphones have often been hailed as opportunity to reach a more diverse sample. Smartphone usage is widespread: in the UK it is 98% for people between 16 and 44 years, 95% between 45 and 54 years and 87% for people between 55 and 64 years [76]. The ability to enrol participants from distance and collect data from distance, remove geographic barriers to participation [68].

Indeed, some smartphone studies have succeeded in recruiting participant groups that traditionally are harder to reach. This includes younger, more active participants, people with mental illnesses, people living in rural locations, people with severe progressive diseases [77–79]. In addition, smartphones have been shown useful for data collection during the COVID-19 pandemic, when clinical data collection halted [80]. Furthermore, previous smartphone studies have recruited large numbers of participants from distance - in the order of magnitude of thousands of participants - by instructing them to download an app and register online [17, 44, 45, 65].

### Reality: smartphone studies may not succeed in recruiting representative participant groups and impose barriers to participation

When people are recruited for smartphone studies in clinic, ‘smartphone ownership’ or ‘smartphone usage’ is often an inclusion criterium. When people are enrolled from distance, this often means that ‘digital literacy’ is an implicit prerequisite: only people that succeed in downloading an app and registering, will succeed in participating in a study.

These inclusion criteria or prerequisites can influence the representativeness of the final study sample. Although smartphones are used widely, their use is less prevalent in certain demographic groups, and in certain parts of the world. Seldom-heard groups may include underprivileged groups [67], people with low socio-economic status [26], people with low literacy skills or low digital literacy [26, 66] and older people [62, 77]. In addition, if participants are recruited remotely, it may be more difficult to validate that participants belong to the target population beyond self-report [20].

The risk of selection bias is especially large if researchers develop an app for one operating system only. The operating systems Android (Google) and iOS (Apple) each cover around half of the smartphone market (Android being used slightly more frequently in low-income countries, and iOS slightly more in high-income countries) [81]. Apps that are developed for one operating system only (such as apps using the Apple Research Kit [77]), will not reach the other half of the population. Both halves are not interchangeable: on average, the annual income of iOS users is almost 50% more than Android users [27].

### Hope: researchers should refrain from generalising their results, and undertake active steps to increase representation of seldom-heard groups

With smartphone studies, it is even more important to limit inferences to the sample population only, rather than extrapolating findings to larger groups. Some limitations to generalizability are intuitive (e.g. absence of the oldest age brackets due to lack of smartphone penetration), but others may be less obvious (e.g. selection of specific income group because of choice of operating system). Researchers should undertake action to reach people from underprivileged groups [67]. In the context of musculoskeletal diseases, this may include people of low socio-economic status and people with multiple morbidities.

### Hype: sensor data is objective

Passive data collection can provide frequent and granular information on exposures, outcomes and covariates (including time-varying confounders), and day-to-day participant behaviour and exposure [17, 33–39, 82–85]. Smartphone sensors increase the range of domains which can be measured in participants’ daily lives, including aspects like time spent at home, minute-to-minute weather exposures and sleep [37, 86, 87]. In addition, smartphone sensors can improve the accuracy of reporting: passive data is not plagued by the information biases of self-reported data, such as social desirability bias (e.g. people under-reporting drug use or sedentary time [88, 89]) or recall bias (e.g. people misremembering past exposures). It is therefore often called ‘objective’ and hailed as gold standard for measuring various constructs.

### Reality: sensor data and any metrics derived from it are subject to researchers’ choices and can still be biased

However, converting sensor data into summaries of exposures, outcomes and behaviours is still subject to choices made during data analysis. For example, algorithms for human activity recognition require a multitude of choices during pre-processing (dividing time series into smaller epochs; the number and type of features that are extracted from raw data; the statistical method chosen for activity classification) [90]. A review of smartphone studies using sensor data showed that it can be difficult to assess the ground truth for sensor data, and that some studies found little relation between sensor data and validation measures [37]. Furthermore, many algorithms are developed and tested in a laboratory, and generalize poorly to free-living settings [90]. When these algorithms are applied to real-life data, results may be far from accurate or objective. Algorithms from commercial devices are known to introduce substantial bias when applied in free-living settings (especially if the population of interest is different from the testing population, which often comprises the specific demographic of thirty-year-old healthy males) [91].

Second, raw sensor data underlying these analyses can be subject to different biases. In ‘bring-your-own-device’ studies participants use their personal smartphones, which may be from different brands, model types and software, potentially with defects to screens or sensors [32, 92]. The software used can, for example, influence the amount of missing sensor data [93]. In addition, smartphones often show heterogeneity in accelerometry and gyroscopy data, large enough to potentially influence results [94]. Of note, these technological factors change over time and are difficult to influence [95]. In addition, they can both differ between participants, and within a participant over time.

Third, participant behavior can influence the amount of data collected, the quality of data or the accuracy of summary metrics. For example, accelerometry-based markers of physical activity depend on the position of the smartphone. Algorithms that assume that a smartphone is pocket-worn do not perform as well on data from bag-worn smartphones [96]. Currently, it is still difficult to ascertain whether a smartphone is carried in a position appropriate for the event being sensed [36, 96]. As there are systematic differences in phone use between participants - women tend to carry it mostly in a bag whereas men carry it in their pocket [97] - this may cause misclassification or bias.

Fourth, conversion of sensor data in meaningful summary statistics may introduce specific biases in the musculoskeletal context. Studies have shown that algorithms that perform well in healthy volunteers, often do not perform well in people with musculoskeletal conditions, who tend to walk slower and have different gait characteristics [98, 99].

Fifth, sensor data may not always be available. For example, location data is not available if participants switch their phones off, and may not be available if the phones are indoors or out of battery [27, 78, 93, 100]. In addition, data may be missing by design: for data streams that are battery-intensive, it is not feasible to collect data continuously [22, 27, 37, 69]. Missing sensor data is often not missing at random, and may be related to the participants’ exposure status or outcome status [63].

### Hope: the analysis of sensor data will be increasingly transparent, validated in all patient groups and include uncertainty quantification

Researchers should be transparent about the choices they make during the processing of sensor data, for example by sharing source code [90]. Proprietary apps and algorithms are less suitable for academic research, which requires providing the details needed for reproducibility. Algorithms should be validated against gold standards, in patient groups as well as in healthy volunteers. In some cases, researchers might do well to use such a research-grade wearable rather than a smartphone for sensor data collection, for example, if performance of the former is better in people with musculoskeletal conditions.

To improve the accuracy of mobility metrics, researchers could collect location data alongside accelerometer data, although this comes at a cost to privacy [41, 75]. Some research smartphone apps, however, provide the possibility to (a) record distances rather than locations, or (b) add Gaussian noise to location data to de-identify data, providing higher accuracy while preserving privacy [75].

Furthermore, research efforts should be diverted to algorithms for uncertainty quantification. Any point estimate of someone’s step count is unlikely to be the truth (‘you took 8721 steps today’). Algorithms should therefore provide a 95% confidence interval, ideally considering the amount of missing data and considering the position of the smartphone on the participants’ body [90]. For participants with complete data and a body-worn smartphone, this confidence interval would be much narrower than for participants with high amounts of missing data, or who had their phone lying on a desk. If accuracy and validity is insufficient, researchers should consider using body-worn devices, such as wearable activity trackers [101] or smartwatches [22].

### Hype: smartphone research is cheap

Smartphone studies are often argued to be a cheap option for data collection, as they enable remote enrolment, remote data collection, and participants can use their own device and phone subscription or WiFi network. These bring-your-own-device studies can remove the need for enrolment events and clinic visits for data collection [22, 35]. This reduces costs associated with staffing costs during recruitment or data collection, costs for consumables or physical storage space. Off-the-shelf smartphone apps can be tweaked to specific studies for as little as £1000 to £30,000; other apps are freely available under an open source license [27, 77]. Once an app has been developed, the marginal costs of enrolling an additional 1000, 10,000 or even 100,000 participants is low [36, 77, 102]. As a result, the marginal costs of using a smartphone app for data collection may be lower than those of using paper-based data collection [58].

### Reality: smartphone research can be expensive

Although smartphone studies reduce some costs compared to traditional studies, they create new, often hidden costs. First, app development is not necessarily cheap. Development of a bespoke research app requires collaboration with software engineers and UX designers. It can be time-consuming, since app development usually entails an iterative approach, co-development with patients and careful piloting in feasibility studies [18, 32, 54, 69, 103]. Feasibility studies are essential to determine if the target population finds the app easy to use, balance data collection needs with preservation of battery life of participants’ devices, and determine the optimal frequency of active and passive data [32, 69, 79]. Modification of off-the-shelf apps or platforms can be simpler, although these may come with licensing fees.

Second, the use of smartphone apps may require research budget towards data storage [27, 32, 77], licenses for data analysis software, and, especially if high volume sensor data is collected, computing infrastructure [27].

Third, smartphone studies require maintenance and support. Smartphone models and smartphone software (i.e. the operating systems Android and iOS) are frequently updated and these updates can delay or block data collection [27, 32]. Costs associated with such updates can be substantial and can be, for example, in the range of $100,000 per year. In addition, technical support may be required in case participants face problems with the app.

### Hope: smartphone studies will be cost-effective and efficient

It would be helpful if the musculoskeletal research community could share expected and unexpected costs associated with smartphone studies. Cost comparisons between studies enable researchers to identify what types of apps and infrastructures fit their budget. If researchers report costs per expense (e.g. app development, maintenance, storage, analysis; separating costs of staff time from work by external parties from costs of hardware or software) as well as marginal costs per participant, the research community can identify sources of variability in costs, as well as areas where improvement would lead to the highest reduction in costs.

Secondly, we believe that smartphone studies would be more efficient if they could use shared platforms. Examples of app platforms that are suitable for research are Beiwe (open source/non-commercial [27, 92],), uMotif [17, 21, 42] and Apple ResearchKit [68, 77]. Re-use of high-quality platforms will prevent researchers from reinventing the wheel, and from developing apps that are unsafe, unsecure or unwell engineered [103]. Especially open source software should be of interest of the research community. Open source apps can be reviewed by an unlimited base of software engineers and improve reproducibility and transparency of studies [27, 68]. In addition, researchers can develop extensions or new features and contribute the code so that other groups can use those extensions too.

Third, research apps could provide more utility when clinically implemented and linked with electronic medical records [104]. Data from research apps could transform consultations for clinician and patient benefit and aid shared decision-making [6, 105]. Furthermore, such applications of research apps could open doors to new funding opportunities. However, there are few published efforts on efficacy, effectiveness and feasibility [105]. Integration of research data into the electronic medical record also requires overcoming various barriers, including issues around sharing, privay and governance [18, 26].

## Conclusions

Without doubt, smartphone studies represent an exciting and rapidly growing area of research development. In this article, we provided an insight in the plethora of benefits around size, scale and frequency of data collection. In musculoskeletal research, smartphones provide special benefits, as the target group face chronic conditions (increasing the importance of long-term data collection), characterised by symptoms that affect mobility and physical activity (potentially easier to measure with smartphones) as well as a range of patient-reported outcomes (self-reported more frequently at lower burden to the participant). We discussed various studies that showcased the unique benefits from smartphones in the musculoskeletal context.

However, despite these substantial and exciting benefits, smartphone studies are not free from challenges and do not solve all challenges. If smartphone studies are designed without awareness of the challenges inherent to smartphone use, they may fail or may provide biased results. In this article, we therefore reviewed the known limitations of smartphones and provided lessons for future smartphone studies.

We showed that achieving high engagement of participants may be a challenge, as well as recruiting representative samples, including less privileged people, or people with low digital literacy. We argued that sensor data is by no means objective, even though it removes some biases associated with self-reported exposures and outcomes. Finally, we discussed the costs of smartphone studies, noting that even though participants may bring their own device, smartphone studies can still bring substantial costs of app development and testing, data storage and analysis.

Of note, we conducted a narrative review and did not perform a systematic search of smartphone studies, a systematic quality appraisal of studies or a systematic search of hypes and hopes. The examples that we included are for illustrative purposes, and this narrative review, although hopefully informative to the reader, is unlikely to be comprehensive. Where possible, we have provided references to reviews both of smartphone studies in musculoskeletal conditions (such as [18, 28, 50–54, 56]) and of the hypes and hopes we discussed (such as [20, 36, 39, 66, 89]). These reviews tend to be narrower in scope than our overview, but provide a more comprehensive overview of the subject area and often contain systematic quality appraisals.

We hope the musculoskeletal research community will join us in paving the way forwards. In this journey, transparency is key. Smartphone studies in the healthcare context often do not report essential information on study design (e.g. details on app development or privacy protection [37, 103]), data collection (e.g. the method for location data collection [39]) and data analysis (e.g. the algorithm used to convert sensor data into human activity metrics [90]). Better transparency can be stimulated through the use of reporting guidelines. For smartphone-based interventions, two checklists provide reporting guidelines, one for trials using web-based health interventions (CONSORT-EHEALTH statement [106]) and one for mobile phone-based health interventions (the mERA checklist [107]). For apps aimed at self-management by people with muskuloskeletal disorders, an EULAR task force articulated three overarching principles and ten points to consider [108]. Various reporting guidelines directly relate to the hypes and hopes discussed in this article. For example, the mERA requirement of providing a cost assessment of the intervention would fulfil our hope of more insights in the costs and savings associated with smartphone studies. Wider adoption of the best practices described in these guidelines will contribute to transparency. Transparency will help prevent others to re-invent the wheel and to contribute to cost-effective and efficient smartphone studies. Regardless of available reporting checklists, we encourage full and comprehensive sharing of app development, piloting procedures and research methods.

Awareness of the challenges of smartphone studies will help researchers anticipate or avoid common pitfalls. In addition, it would be useful to bring the musculoskeletal research community together to exchange lessons learnt on issues specific to our field. These issues may include symptom-related barriers to using smartphones for research, validating algorithms in patient populations with reduced functional ability, digitising validated questionnaires, and methods to reliably quantify pain, quality of life and fatigue. We recommend that researchers share both the successful and unsuccessful strategies they employed for others to learn from. We hope this review will support researchers to generate more success stories of smartphone studies in musculoskeletal research.

## Data Availability

Not applicable.

## Abbreviations

CONSORT-EHEALTH: Consolidated Standards of Reporting Trials of Electronic and Mobile HEalth Applications and onLine TeleHealth
EULAR: European Alliance of Associations for Rheumatology
mERA: Mobile health (mHealth) evidence reporting and assessment
